# A descriptive study on multisystem inflammatory syndrome in children in a single center in West Michigan

**DOI:** 10.1186/s12969-021-00658-3

**Published:** 2021-12-16

**Authors:** Jonathan Shabab, Allysen Dubisky, Ambaris Singh, Megan Crippen, Khalid Abulaban, Aileen Aldrich

**Affiliations:** 1 Spectrum Health Helen DeVos Children’s Hospital/Michigan State University Pediatric Residency Program, Grand Rapids, MI USA; 2 Michigan State University College of Human Medicine, Grand Rapids, MI USA; 3 Department of Pediatric Rheumatology, Spectrum Health Helen DeVos Children’s Hospital, Grand Rapids, MI USA; 4 Department of Pediatric Infectious Diseases, Spectrum Health Helen DeVos Children’s Hospital, 35 Michigan St. NE, Suite 4150, Grand Rapids, MI 49503 USA

**Keywords:** MIS-C, Multisystem inflammatory syndrome in children, Pediatrics, COVID-19, SARS-CoV-2, Anakinra

## Abstract

**Background:**

Multisystem Inflammatory Syndrome in Children (MIS-C) is a rare hyperinflammatory condition that occurs following SARS-CoV-2 infection. There is a paucity of research describing risk factors, optimal management, and outcomes of this life-threatening condition.

**Methods:**

This is a case series of 26 patients diagnosed with MIS-C in a West Michigan pediatric tertiary care center from April 2020 to February 2021. We describe the clinical, imaging, and laboratory characteristics of these patients and detail their treatments and outcomes with comparisons between Pediatric Intensive Care Unit (PICU) and non-PICU patients. Categorical testing utilized Chi-square and Fisher’s Exact tests. Comparison between groups used T-tests or Kruskal-Wallis.

**Results:**

Fifteen patients (57%) required intensive care. There was no statistically significant difference in demographics between PICU and non-PICU patients, however all Black patients required intensive care. Gastrointestinal symptoms were present in 22 patients (84%). Seventeen patients (65%) had Kawasaki-like features and 12 (46%) developed coronary artery dilation. Patients requiring intensive care were less likely to have a reported history of COVID-19 disease or exposure (*p* = 0.0362). Statistically significant differences were also noted in peak ferritin (*p* = 0.0075), procalcitonin, and BNP in those who required intensive care.

**Conclusions:**

Although overlap exists with other hyperinflammatory conditions, our study provides further evidence that MIS-C is a distinct, albeit heterogenous, disorder with various degrees of cardiac involvement. Anakinra, in conjunction with steroid use, appears to be effective and safe in the treatment of MIS-C. This report identifies procalcitonin, peak ferritin, and BNP as potentially useful biomarkers for severity of disease.

**Supplementary Information:**

The online version contains supplementary material available at 10.1186/s12969-021-00658-3.

## Background

In late 2019, a cluster of viral pneumonia cases of novel coronavirus etiology was noted in Hubei Province, China, with the virus subsequently identified as SARS CoV-2 [1]. Early on, advanced age was recognized as a major risk factor for disease severity and death [2, 3]. Conversely, children appeared relatively spared [4, 5]. However, as SARS-CoV-2 spread across the world into a global pandemic [1], London researchers recognized a new entity [6], originally described as a severe Kawasaki-like disease noted in children across Europe, including Italy and the United Kingdom [7, 8]. This later became known as Multisystem Inflammatory Syndrome in Children (MIS-C) in the United States [6]. Soon after, the Centers for Disease Control and Prevention (CDC) and the World Health Organization (WHO) developed case definitions for the diagnosis of MIS-C [9, 10].

In typical adult cases of COVID-19, patients present with fever, cough, and elevated inflammatory markers, often worsening in the second week of illness [2, 3]. In severe cases, inflammatory markers continue to rise in association with acute respiratory distress syndrome and sepsis, with correspondent morbidity and mortality [3]. Children have been found to have a far lower risk of developing acute respiratory disease requiring hospitalization and intensive care, with the exception of children with specific underlying medical conditions such as obesity [11, 12]. Nevertheless, children are at greater risk of the post-infectious hyperinflammatory disease 2–8 weeks following asymptomatic or symptomatic infection with SARS-CoV-2 [13]. In accordance with the aforementioned case definitions, those with MIS-C have a persistent fever, at least two organ systems affected, and elevated inflammatory markers without alternate explanation [9, 10], although most patients have at least 4 organ systems involved [14]. Approximately 50% of MIS-C patients have cardiac involvement severe enough to require vasoactive or vasopressor support, and in one study of 186 children, 4 died [14].

By February 28, 2021, there were nearly 625,000 confirmed cases of COVID-19 in Michigan [15]. Further, there were 99 reported cases of MIS-C by CDC criteria in the state [9, 16]. At our institution, we developed a multidisciplinary protocol developed to assist in the diagnosis, evaluation, and treatment of MIS-C, modeled from American College of Rheumatology (ACR) guidelines [13]. Patients suspected of having MIS-C are evaluated by pediatric infectious disease and pediatric rheumatology in addition to the pediatric hospitalist and/or intensivist teams. Initial labs and work-up were obtained in accordance with our institutional algorithm (see details in Methods section below and Additional file 1: Appendix A). During hospitalization, MIS-C patients are routinely monitored with daily inflammatory, cardiac, and coagulation labs. Additional subspecialty consults were provided as indicated. Our treatment consists of supportive care, anticoagulation with aspirin, low-molecular weight heparin, or heparin, and immunomodulatory agents including intravenous immunoglobulin (IVIG), steroids, and anakinra, as determined by the multidisciplinary team. Here, we report the clinical aspects, patterns, and outcomes observed at our institution between April 2020 and February 2021 while caring for children with MIS-C.

## Methods

This study was a case series at Spectrum Health Helen DeVos Children’s Hospital (HDVCH), a 241-bed children’s tertiary care center in Grand Rapids, Michigan, USA. Subjects were pediatric patients admitted to HDVCH between April 1, 2020, and February 28, 2021, with a diagnosis of MIS-C as made by the consulting teams in concordance with CDC case definitions. The study received a HIPAA waiver from the Spectrum Health Institutional Review Board.

Once identified as a possible case of MIS-C by clinical criteria, patients were evaluated with complete blood count with manual differential, comprehensive metabolic panel (CMP), as well as inflammatory markers (erythrocyte sedimentation rate [ESR], C-reactive protein [CRP], D-dimer, fibrinogen, and ferritin). Urinalysis was obtained to evaluate for sterile pyuria and identify alternative causes for fever and inflammation. COVID-19 testing obtained included nasopharyngeal PCR and serum immunoglobulin G (IgG), which at first was a total antibody anti-nucleocapsid assay and later included anti-spike protein IgG. Brands and versions of the PCR and IgG tests used evolved over time based on test availability and performance. Any patients admitted to the hospital with suspicion for MIS-C underwent further laboratory evaluation, including coagulation studies (prothrombin time [PT], internationalized normal ratio [INR], and partial thromboplastin time [PTT]), procalcitonin, triglycerides, lactate dehydrogenase (LDH), creatinine kinase, and peripheral blood smear. If initial COVID-19 PCR testing was negative, a full respiratory pathogen panel (if not already performed), blood culture, and urine culture were performed to identify alternative etiologies. Patients admitted to the hospital with suspicion of MIS-C had daily labs, trending complete blood counts with manual differential, CMP, ESR, CRP, D-dimer, ferritin, and fibrinogen.

Initial cardiac evaluation for patients presenting with possible MIS-C included EKG and high sensitivity troponin. Following admission, patients were evaluated with brain natriuretic peptide (BNP) levels, and an echocardiogram was performed. BNP and troponin were repeated daily during admission. Echocardiograms were repeated based on clinical features, whether an alternate diagnosis was made, or as recommended by pediatric cardiology. Patients with cardiac abnormalities were followed by pediatric cardiology both during and after hospitalization with serial imaging.

Comparisons were performed on PICU versus non-PICU patients as a surrogate for disease severity. All statistical analyses were completed using SAS Enterprise Guide version 7.1 (SAS Institute Inc., Cary, NC, USA). Normality was assessed using a Shapiro Wilk test. Differences in categorical data were analyzed using Chi-square tests or Fisher’s-Exact tests. For normally distributed and continuous data, means and standard deviation were described and T-tests were used to evaluate for significant differences. For non-normally distributed and continuous data, medians and interquartile values were established and Kruskal-Wallis tests were used to compare medians. For all analyses, *p* values of < 0.05 were considered significant.

## Results

**Fig. 1:**
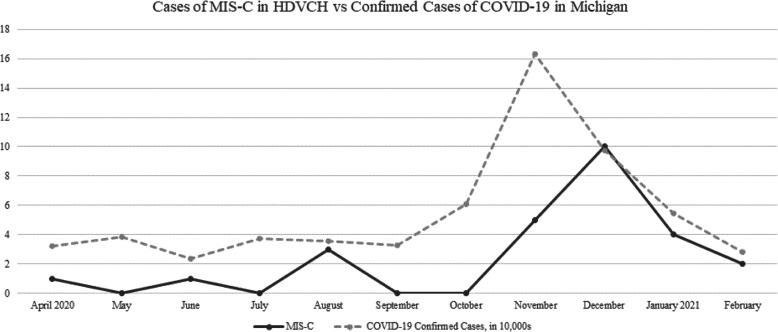
Comparison of MIS-C Cases and COVID-19 Cases by Month, 2020–2021. The above graph depicts the total number of cases of Multisystem Inflammatory Syndrome in Children (MIS-C) by month for our institution, Helen DeVos Children’s Hospital (HDVCH) (solid line), along with the total number of reported COVID-19 cases within the state of Michigan (dashed line) during this same time frame (note this data is in tens of thousands). This includes cases diagnosed/reported according to CDC and/or WHO criteria from April 1, 2020, through February 28th, 2021. Note the spike in MIS-C cases approximately 1 month after the COVID-19 spike. Source: https://www.michigan.gov/coronavirus/0,9753,7-406-98163_98173_104661---,00.html

There were 26 children diagnosed with MIS-C associated with COVID-19 at our institution between April 1, 2020, and February 28, 2021, which accounted for about one-third of the total cases in our state (Fig. 1). Of these, 15 (57%) children required admission to the pediatric intensive care unit (PICU) designated as group 1. Those who did not require care in the PICU were designated as group 2. No statistically significant differences were noted in patient demographics which are delineated in table one. The clinical characteristics and cardiac findings of these 26 children are outlined in Table 1. Three patients had diagnosis of obesity prior to admission.

**Table 1:** Clinical characteristic and cardiac findings in patients with Multisystem Inflammatory Syndrome in Children in Michigan stratified by ICU admission, April 2020–February 2021

Demographic Characteristics					
		Total (%)	Group 1 (PICU) (%)	Group 2 (non-PICU) (%)	*p* values
Total		26	15	11	
Age (months)	Mean (St Dev)	105.15 (57.3)	99.13 (53.4)	113.36 (64.0)	0.5424
Sex	Male	16 (61.5)	7 (46.7)	9 (81.8)	0.1092
	Female	10 (38.5)	8 (53.3)	2 (18.2)	
Race	White	14 (53.5)	8 (53.3)	6 (54.6)	–
	Black	4 (15.4)	4 (26.7)	0 (0)	–
	Hispanic	3 (11.5)	2 (13.3)	1 (9.1)	–
	Other	3 (11.5)	0 (0)	3 (27.3)	–
	Unknown	2 (7.7)	1 (6.7)	1 (9.1)	–
BMI	Median (IQR)	18.095 (16.1–20.83)	17.7 (16.1–20.06)	19.16 (15.44–22.98)	0.5858
Clinical presentation					
Fever		26 (100)	15 (100)	11(100)	1.0
GI symptoms	Vomiting	19 (73.1)	12 (80)	7 (63.6)	0.4065
	Diarrhea	14 (53.9)	7 (46.7)	7 (63.6)	0.3912
	Abdominal pain	22 (84.6)	14 (93.3)	8 (72.7)	0.2789
Skin rash		15 (57.7)	8 (53.3)	7 (63.6)	0.7007
Other Kawasaki-like features^a^		17 (65.4)	8 (53.3)	9 (81.8)	0.1315
Cardiac involvement	Coronary dilation	12 (46.2)	6 (40)	6 (54.6)	0.4623
	Other cardiac changes	15 (57.7)	11 (73.3)	4 (36.4)	0.1089
History of COVID-19 infection/exposure		17 (65.4)	7 (46.7)	10 (90.9)	0.0362*
Cytokine elevation^b^	IL-2 Soluble	8 (50)	4 (40)	4 (66.7)	0.6084
	IL-2 Receptor	12 (75)	9 (90)	3 (50)	0.1181
	IL-5	2 (12.5)	2 (20)	0 (0)	0.5
	IL-6	7 (43.8)	6 (60)	1 (16.7)	0.1451
	IL-10	16 (100)	10 (100)	6 (100)	
	IL-13	6 (37.5)	5 (50)	1 (16.7)	0.3069
	IL-17	5 (31.3)	4 (40)	1 (16.7)	0.5879
	TNF-alpha	3 (18.8)	2 (20)	1 (16.7)	1.0
	TNF-gamma	6 (37.5)	4 (40)	2 (33.3)	1.0
Positive COVID-19 PCR on admission		11 (42.3)	6 (40)	5 (45.5)	1.0
COVID-19 antibody	Anti-Spike	25 (96.2)	15 (100)	10 (90.9)	0.4231
	Anti-Nucleocapsid	18 (85.7)	10 (83.3)	8 (88.9)	1.0
Respiratory support	Nasal cannula	2 (7.7)	2 (13.3)	0 (0)	–
	BiPap	2 (7.7)	2 (13.3)	0 (0)	–
	Intubated	8 (30.8)	8 (53.3)	0 (0)	–

*BMI* Body mass index, *IQR* interquartile range, *GI* Gastrointestinal, *COVID-19* Coronavirus disease 2019, *PCR* polymerase chain reaction, *IL* Interleukin, *TNF* Tumor necrosis factor, *BiPap* Bilevel positive airway pressure

^a^ cervical adenopathy, oral mucosa changes, peripheral extremity changes, and/or bilateral non-purulent conjunctivitis

^b^ Incorporated into practice during study period, not universally obtained on all included patients

*significant *p* value (≤0.05)

Every patient presented with fever, in accordance with initial diagnostic criteria. Gastrointestinal symptoms were common among these children, with 22/26 (84.6%) experiencing abdominal pain, including 14/15 (93.3%) of PICU cases. Kawasaki-like features (defined as having one or more of the major diagnostic clinical criteria of Kawasaki disease, namely bilateral conjunctivitis, unilateral lymphadenopathy of 1.5 cm or greater, rash, oral mucosa changes, or extremity changes) were seen in 17/26 children (65.4%), 9 of which did not require PICU admission. Coronary dilation (Z-scores ≥2.0 on echocardiogram) was seen in 12/26 children (46.2%), 6 of whom required intensive care and 6 of whom did not. Dilation was seen in the left main coronary, circumflex, left coronary, left anterior descending and right coronary arteries. Other cardiac changes were seen in 15/26 children (57.7%), which included depressed left and/or right ventricle systolic function, mitral and tricuspid regurgitation, aortic root dilation, main pulmonary artery enlargement and pericardial effusion.

There were 17 children with a known history of COVID-19 infection or exposure; of these, 7 were in the PICU group and 10 in the non-PICU group, meaning patients in the PICU were less likely to have a COVID-19 history (*p =* 0.0362). The COVID-19 anti-spike antibody was found in nearly all children (25/26, 96.2%). Only one patient lacked anti-spike antibodies, and this patient was in the non-PICU group. Among the 16 children who had cytokine evaluations (10 in the PICU group, 6 in the non-PICU group), 100% had elevation in IL-10. The PICU group included all children who needed any respiratory support, typically related to cardiorespiratory compromise (12/26, 46.2%). There were 2/15 (13.3%) who required nasal cannula, 2/15 (13.3%) who required bilevel positive airway pressure, and 8/15 (53.3%) who required intubation amongst all the PICU cases. Multiple imaging studies were ordered on the patients in this study depending on their clinical symptoms; details and statistical analysis were beyond the scope of this review.

All 26 patients underwent EKGs, 20 (76%) of which were abnormal. The abnormal EKG findings included sinus tachycardia, sinus bradycardia, nonspecific ST segment and T wave changes, low voltage QRS complexes, poor R wave progression, prolonged QT interval, borderline prolonged PR interval, right axis deviation, left axis deviation, premature ventricular contractions, and possible right ventricular hypertrophy.

Laboratory data at presentation are outlined in Table 2. All patients had elevated inflammatory markers at presentation, which were necessary criteria for diagnosis of MIS-C. Other general lab findings included anemia, lymphopenia, elevated creatinine, mild elevation in AST and/or ALT, and elevated troponin and BNP levels. The PICU group had significantly higher procalcitonin levels with a median of 11.1 ng/mL while the non-PICU group had a median of 2.79 ng/mL (*p* = 0.0298). The BNP level was significantly higher in the PICU group with a median of 7019 pg/mL compared to the non-PICU group with a median of 1179 pg/mL (*p* = 0.0218). The ferritin level at presentation, although higher in the PICU group, was not statistically significantly higher than in the non-PICU group; however, the peak ferritin level in the PICU group was significantly higher than the non-PICU group (median 1008 vs 270, *p* = 0.0075). The mean hemoglobin level in the PICU group was significantly lower at presentation than the non-PICU group (10.31 g/dL vs 12.05 g/dL, *p* = 0.0196). The median AST for the PICU group was higher than the non-PICU group (39 U/L vs 22 U/L, *p* = 0.0331). The non-PICU group had higher LDH and CK medians than the PICU group, though not statistically significant.

**Table 2:** Laboratory data at presentation for patients with Multisystem Inflammatory Syndrome in Children in Michigan, April 2020 – February 2021

	Total	Group 1 (PICU)	Group 2 (non-PICU)	Reference value	*p* values
Total	26	15	11		
WBC (K/CUMM)	10.385 (8.76–13.03)	10.70 (8.76–14.46)	9.51 (7.65–11.6)	5.00–15.00	0.4062
Hgb (g/dL)^a^	11.04 (1.93)	10.31 (1.84)	12.05 (1.62)	11.5–14.5	0.0196*
Platelets (K/CUMM)	179.5 (137–226)	147 (124–209)	183 (158–250)	140–400	0.2128
ALC (K/CUMM)	880.5 (402–1490)	876 (299–1490)	1073 (580–1866)	800–7900	0.3781
Sodium (mMol/L)^a^	133.73 (5.44)	134.27 (6.53)	133 (3.66)	134–146	0.5685
Bicarbonate (mMol/L)	22 (19–24)	22 (19–24)	24 (19–24)	21–29	0.4951
Creatinine (mg/dL)	0.475 (0.33–0.70)	0.48 (0.32–0.70)	0.41 (0.33–0.76)	< 0.6	0.8762
Albumin (g/dL)^a^	2.76 (0.64)	2.62 (0.69)	2.95 (0.54)	3.5–5.0	0.2070
ALT (U/L)	26 (16–39)	27 (16–51)	25 (13–37)	< 40	0.5853
AST (U/L)	32 (21–44)	39 (25–70)	22 (17–39)	< 40	0.0331*
LDH (U/L)	280.5 (222.5–341)	267 (217–334)	327 (228–348)	< 271	0.4516
Triglycerides (mg/dL)	131 (103–185)	130 (102–185)	132 (124–153)	< 150	0.8053
ESR (mm/h)^a^	48.12 (30.4)	44.33 (29.59)	53.27 (32.16)	< 13	0.4701
CRP (mg/L)	110.3 (82.6–164.9)	145 (95.1–177.9)	90.9 (76–110.3)	< 5	0.0616
Procalcitonin (ng/ml)	6.74 (2.78–16.84)	11.1 (5.7–32.8)	2.79 (1.52–4.98)	< 0.25	0.0298*
Ferritin (ng/mL)	374 (178–809)	520 (178–1590)	200 (178–604.2)	< 336	0.1611
Peak Ferritin (ng/mL)	690.5 (274–1214)	1008 (513–1733)	270 (200–829)	7–142	0.0075*
D-dimer (mg/l FEU)	2190 (1030–3800)	2355 (1540–6850)	2190 (690–2810)	< 0.5	0.2983
BNP (pg/mL)	2147 (552–11,023)	7019 (1631–20,170)	1179 (245–2147)	< 101	0.0218*
Troponin (ng/L)	18 (5.99–40)	18 (5.99–40)	13 (5.99–45)	< 17	0.6739
CK (U/L)	73.5 (43–215)	59 (43–268)	88 (24–162)	< 233	0.5518
Fibrinogen (mg/dL)^a^	505.27 (127.51)	496.27 (129.8)	517.55 (129.51)	190–400	0.6830
PTT (s)	26 (23.5–28)	26 (24–29)	26.5 (23–27)	< 33.1	0.9765
INR	1.1 (1.1–1.2)	1.2 (1.1–1.6)	1.1 (1–1.2)	< 1.1	0.1121

*IQR* Interquartile range, *WBC* White blood cells, *Hgb* Hemoglobin, *ALC* Absolute lymphocyte count, *ALT* Alanine aminotransferase, *AST* Aspartate aminotransferase, *LDH* Lactate dehydrogenase, *ESR* Erythrocyte sedimentation rate, *CRP* C-reactive protein, *BNP* Brain natriuretic peptide, *CK* Creatine kinase, *PTT* Partial thromboplastin time, *INR* International normalized ratio

^a^These were normally distributed data, therefore mean and standard deviations are reported. The remainder of the data was not normally distributed and so medians and interquartile ranges are reported

*significant *p* value (≤0.05)

The management of the 26 patients with MIS-C is outlined in Table 3. There were 25 children who received aspirin and 25 children who received IVIG (2 g/kg), with only one child in the non-PICU group not receiving either due to a very mild clinical case. Severe presentations or those not clinically responsive to IVIG were additionally treated with IV methylprednisolone, either low dose (2 mg/kg/day as a single dose or divided twice a day) or pulse dosing (15-30 mg/kg with maximum of 1000 once daily for 3–5 days). Corticosteroids were given to 22 children, 13 of whom were in the PICU. Seven children received pulse corticosteroids, only 1 of whom was outside the PICU. Anakinra was given more frequently in the PICU group compared to the non-PICU group (66.7 vs 27.3%, *p* = 0.0472).

**Table 3:** Management of 26 patients with Multisystem Inflammatory Syndrome in Children in Michigan, April 2020 – February 2021

Treatment				
	Total (%)	Group 1 (PICU) (%)	Group 2 (non-PICU) (%)	*p* values
Total	26	15	11	
Inotropes^a^	12 (46.2)	12 (80)	0 (0)	0.0001*
Aspirin	25 (96.2)	15 (100)	10 (90.9)	0.4231
Low-molecular weight heparin	12 (46.2)	9 (60)	3 (27.3)	0.0982
Heparin	5 (19.2)	5 (33.3)	0 (0)	0.0527
IVIG	25 (96.2)	15 (100)	10 (90.9)	0.4231
Steroids	22 (84.6)	13 (86.7)	9 (81.8)	1.0
Steroid pulse	7 (26.9)	6 (40)	1 (9.1)	0.1783
Anakinra	13 (50)	10 (66.7)	3 (27.3)	0.0472*
Infliximab	1 (3.9)	1 (6.7)	0 (0)	1.0

*IVIG* Intravenous immune globulin

^a^This included epinephrine, norepinephrine, vasopressin, and/or milrinone

*significant *p* value (≤0.05)

Of those admitted to intensive care, 12 out 15 (80%) required inotropes (epinephrine, norepinephrine, vasopressin, and/or milrinone). Anticoagulation with low molecular weight heparin or heparin was given to 9 of 12 children (60%) in the PICU group and 5 children in the non-PICU group; all 5 children who received heparin were in the PICU group. Infliximab was given to one child (3.9%) that was in the PICU group and was diagnosed with MIS-C retrospectively based on clinical characteristics but at the time (April 2020) was treated as Kawasaki Shock Syndrome. One patient received antiviral treatment with remdesivir due to concern for concurrent acute COVID-19 infection and marked severity of illness who subsequently expired.

Concurrent acute bacterial infections occurred in 2 patients (8%), including a urinary tract infection in one and another with a central line-associated bloodstream infection complicated by peritonitis. Other complications included extracorporeal membrane oxygenation (ECMO) use in 3 patients (12%), ischemic cerebrovascular accident or intracranial hemorrhage in 2 patients (8%), bilateral upper and lower extremity amputations in 1 patient (4%), persistent first-degree atrioventricular (AV) block in 1 patient (4%), requirement of enteral tube feeds at discharge in 1 patient (4%), disseminated intravascular coagulation in 1 patient (4%), and death in 1 patient (4%). Median length of stay was 6 days (range 2–43 days) overall, with longer admissions for those in the PICU group compared to the non-PICU group (8 days vs 5 days, *p* = 0.002).

All discharged patients had pediatric specialty follow-up with cardiology, infectious diseases, and rheumatology. Patients treated with corticosteroids completed a 2–6 week oral taper after discharge as per ACR guidelines; weans were performed at the discretion of the treating rheumatologist in light of relevant clinical or laboratory abnormalities. The duration of anakinra varied widely from only a few days during their inpatient stay to several weeks past discharge. Increased institutional experience in association with reports from other groups over time allowed for reduction of total duration of anakinra therapy. One patient required readmission for recrudescent fevers and increase in inflammatory markers after completing a 6-week oral corticosteroid taper. It is worth noting that the initial presentation was consistent with MIS-C. Anakinra therapy was continued for 3 months. Trial off resulted in clinical and laboratory recurrence of inflammatory features. Subsequently she transitioned to canakinumab (IL-1 antagonist) and is currently clinically well. This patient did undergo further investigative work up looking for potential other autoinflammatory disease which was non-diagnostic and has whole genome sequencing studies pending.

## Discussion

### Overall findings

From a demographic standpoint, ethnicity and race have been reported to impact the severity of disease in those affected by acute respiratory COVID-19 due to a variety of proposed mechanisms [17, 18]. The same effect of race/ethnicity on disease severity has not yet been demonstrated in MIS-C. In this report, we did not find a statistically significant difference in demographics and outcomes. However, it should be noted that all Black patients included in this study were admitted to the PICU, and that this group included the only mortality as well as the patient with the most significant morbidity (amputations). One unusual finding in comparing the PICU vs non-PICU group features is the lower incidence of a reported prior COVID-19 history in patients admitted to the PICU. Whether this relates to more rapid immune dysregulation [19, 20], delayed presentation to care, or concomitant acute manifestations of COVID-19 is unclear and further studies with larger cohorts are needed.

### Lab differences

Statistically significant differences were found between the PICU and non-PICU groups regarding laboratory values of hemoglobin, BNP, AST, peak ferritin, and procalcitonin. These laboratory derangements correlated with disease severity in our cohort.

Mean hemoglobin was found to be lower in the PICU group; however, the interpretation of this finding is uncertain, especially given that normal ranges for hemoglobin vary by age. Anemia has been frequently described in critical care settings as well in inflammatory states, and the etiology of anemia in our cohort may be multifactorial: iatrogenic (more frequent blood draws, dilution from fluid resuscitation), anemia of acute inflammation, and possibly preexisting anemia of iron deficiency [21]. Our small cohort and retrospective study design do not allow us to delineate between these potential causes. On average, BNP was greater in the PICU group than the non-PICU group. Elevated BNP correlates with greater cardiac dysfunction, and indeed may correlate with disease severity.

In the PICU group, we observed greater elevation in AST, peak ferritin, and procalcitonin. The elevation in AST may be secondary to the greater organ dysfunction and shock present in the PICU group [22]. Elevations in ferritin are commonly associated with inflammatory and immune dysregulation states. This, in combination with the increased AST, is reminiscent of hemophagocytic lymphohistiocytosis and macrophage activation syndrome (HLH/MAS). These immune dysregulation disorders have previously been reported secondary to viral illness [22] and so it may follow that MIS-C may have a similar pathophysiological basis with SARS-CoV-2 as a trigger. Procalcitonin was also substantially elevated in the ICU patients; this may be a biomarker of multi-organ dysfunction, as excretion of procalcitonin is mediated by the kidneys. However, procalcitonin was elevated in patients who did not have documented renal dysfunction, and may therefore be a biomarker for disease severity in MIS-C.

### Treatment approaches

Most patients that underwent treatment for MIS-C received IVIG as per our institutional guidelines. Corticosteroids were used as adjuvant therapy in the majority of patients unless presentation was very mild. Pulse dose corticosteroids were used in patients with critical illness requiring inotropic medication and positive pressure ventilation, or extensive changes on echo including extensive coronary artery dilation or clinically significant effusion. Those with severe presentation, continued decline, or poor response to other therapies were additionally treated with anakinra. Statistically significant differences were found between the PICU and non-PICU groups with usage of inotropes and anakinra. Our institution restricts inotropes to the critical care or emergency department setting, and the majority of the patients cared for in the PICU required inotropic support. We followed the suggestion of early ACR guidelines for anakinra implementation in MIS-C. Our greater reliance on anakinra is likely attributable to the pediatric rheumatology department’s familiarity with using anakinra in managing diseases like systemic juvenile idiopathic arthritis (JIA) [23]. Anakinra is a recombinant interleukin 1 (IL-1) receptor antagonist that is more commonly used to treat autoinflammatory diseases including systemic JIA, cryopyrin-associated periodic fever syndromes and even HLH/MAS [24, 25]. A common pathogenesis of those diseases is an exaggerated innate immune response with the hallmark of an amplified IL-1 cytokine signaling [24, 25].

The principal distinguishing feature of patients who required intensive care was the degree of shock. Our cohort’s patients cared for in the PICU had corresponding elevation in BNP, as well as a hyperinflammatory state demonstrated by elevations in AST, peak ferritin and procalcitonin, which is similar to prior literature [12].

### Outcomes

The majority of patients diagnosed with MIS-C at our institution in the study period were discharged from the hospital in good condition without apparent persistent sequalae. However, several of the patients included in the study group had lingering cardiac disease including coronary dilation, cardiac dysfunction, or both at discharge. We had only one patient with persistent 1st degree AV block after discharge that required prolonged aspirin therapy. The long-term cardiac sequelae of patients diagnosed along the spectrum of severity of MIS-C are yet to be determined [26–28], and chronic follow-up with Pediatric Cardiology is planned for our cohort.

However, some patients did experience substantial morbidity during their hospitalization for MIS-C. In particular, one patient had severe shock and cardiovascular collapse necessitating ECMO. During this time, he developed ischemic injury secondary to edema and disseminated intravascular coagulation leading to dry gangrene of all distal extremities necessitating amputation. He additionally developed profound multiple organ dysfunction and required CRRT, with subsequent hemodialysis. Prior medical history was notable for autism, obesity, and mild intermittent asthma. Unfortunately, our cohort also included one patient death. This patient experienced severe shock with cardiogenic, septic, and distributive features. After a brief period of clinical stability, the patient experienced rapid deterioration and was found to have superimposed gram-negative septicemia. This subsequently led to ECMO due to catecholamine resistant shock. This was complicated by profound intracranial hemorrhage and the patient’s family elected to withdraw technological support. The etiology of the patient’s superimposed bloodstream infection is uncertain, although multiple mechanisms were proposed. It is possible that the patient’s degree of shock led to microangiopathic ischemic changes to the intestinal mucosa that allowed for dissemination of enteric bacteria into the patient’s bloodstream. Further, the patient was substantially immunosuppressed with both pulse and high dose corticosteroids, and anakinra, making her more susceptible to opportunistic infection. Both selected severe cases did occur in Black children.

Notably, as above, our data does suggest that concomitant infections can and do occur in MIS-C. This occurred in two of the patients in our cohort. Whether the hyperinflammatory state predisposes individuals to be at risk of developing other infections or this is subsequent to the effects of immunomodulating medications is unclear. Of note, anakinra has not been associated with secondary infections in prior research on chronic rheumatological conditions [29–31]. Observational studies have demonstrated a dose dependent effect of infection associated with corticosteroid use; however, this has not been replicated with randomized controlled trials [32].

### Addition to the literature

The patients presented in this cohort add to the growing understanding of MIS-C as a potentially life-threatening disease process with a spectrum of cardiac, hematologic and immunologic manifestations. Characteristics of the disease mimic that of other diseases known or believed to be the result of immune dysregulation.

All the patients in our study did have at least some features that overlap with Kawasaki disease, HLH, MAS and other acute immune dysregulation disorders as has been reported in other studies [33]. However, the diagnostic criteria for these conditions were not met in all patients. Therefore, our study adds to the increasing body of evidence that, although apparently related to these other conditions, MIS-C is a distinct disorder with possible overlapping genetic tendencies [34, 35].

Relative to other institutions, our approach to MIS-C generally relied on corticosteroids and anakinra and a collaborative multidisciplinary approach was utilized for diagnosis and management of those patients. For PICU patients, we aimed for complete multidisciplinary rounds daily, when possible, which encouraged real-time discussion of individual cases amongst all involved specialists.

In our study group, no adverse outcomes were noted that were directly attributable to Anakinra use. Given the manner in which we performed our study we cannot directly determine benefit. However, there is a biological plausibility to support its use and accordingly, we believe that randomized controlled trials should be performed.

Some of the laboratory findings in MIS-C that our case series found are reminiscent of an HLH/MAS-like cytokine storm (elevated ferritin, pancytopenia, elevated inflammatory markers). However, the degree of inflammation was not as marked as is typically noted in HLH/MAS, leading credence to MIS-C as a separate disease process with potentially overlapping pathogenesis. Outcomes in our patients were overall favorable while using anakinra which suggests it has a role in management of severe MIS-C when refractory to IVIG and corticosteroids.

### Study limitations

MIS-C is a rare [36] but important syndrome to identify and treat, in light of its widely varying symptomatology and disease severity. Our institution only diagnosed those severe enough to require hospital admission. Our awareness and understanding of the disease evolved as our institutional knowledge increased. This extended to include the diagnostic criteria that were used in conjunction with CDC and WHO guidelines to identify those patients with MIS-C. Given this change in diagnostic criteria it is possible that patients would have been included or excluded differently at different points within the study period.

### Future study

Identification of precise biomarkers that predict severe disease, to quickly identify those patients who may need escalation of care or additional therapeutics, is needed. Although our sample size is low, we suggest that significantly elevated ferritin and procalcitonin may predict a worse disease course.

More research needs to be done to improve our understanding of the patient population that is at the highest risk for developing MIS-C, which remains a rare complication in children after acute COVID-19 infection.

## Conclusion

Although overlaps exist with other hyperinflammatory conditions, our study provides further evidence that MIS-C is a distinct, albeit heterogenous, disorder with possible overlapping features with Kawasaki, MAS, and HLH. Anakinra, in conjunction with corticosteroids, IVIG, and supportive care, is demonstrated to be worthy of evaluation as an adjuvant agent in the treatment of MIS-C. Further, this report identifies procalcitonin, peak ferritin, and BNP as potentially useful markers for severity of disease.

## Supplementary Information

**Additional file 1: Appendix A.** Institutional MIS-C Algorithm.

## Data Availability

The datasets generated and/or analyzed during the current study are not publicly available but are available from the corresponding author on reasonable request.

## Abbreviations

ACR: American College of Rheumatology
AST: Aspartate Aminotransferase
BNP: Brain Natriuretic Peptide
CDC: Centers for Disease Control and Prevention
CMP: Comprehensive Metabolic Panel
COVID-19: Coronavirus Disease 2019
CRP: C-Reactive Protein
ECMO: Extracorporeal membrane oxygenation
EKG: Electrocardiogram
ESR: Erythrocyte Sedimentation Rate
HDVCH: Spectrum Health Helen DeVos Children’s Hospital
HIPAA: Healthcare Information Portability and Accountability Act
HLH: Hemophagocytic Lymphohistiocytosis
IL: Interleukin
IVIG: Intravenous Immunoglobin
JIA: Juvenile idiopathic arthritis
MAS: Macrophage Activation Syndrome
MIS-C: Multisystem Inflammatory Syndrome in Children
PCR: Polymerase chain reaction
PICU: Pediatric Intensive Care Unit
SARS-CoV-2: Severe Acute Respiratory Syndrome Coronavirus 2
WHO: World Health Organization
