# Supplementary material for: A descriptive study on multisystem inflammatory syndrome in children in a single center in West Michigan
Source: Pediatr Rheumatol Online J. 2021 Dec 16;19:172. doi: 10.1186/s12969-021-00658-3 (PMC8675109; doi:10.1186/s12969-021-00658-3)

Appendix A. Institutional MIS-C Algorithm

HDVCH Guideline: Management of Multisystem Inflammatory Syndrome in Children, INPATIENT

Clinical Diagnostic Pathway

Treatment and Management


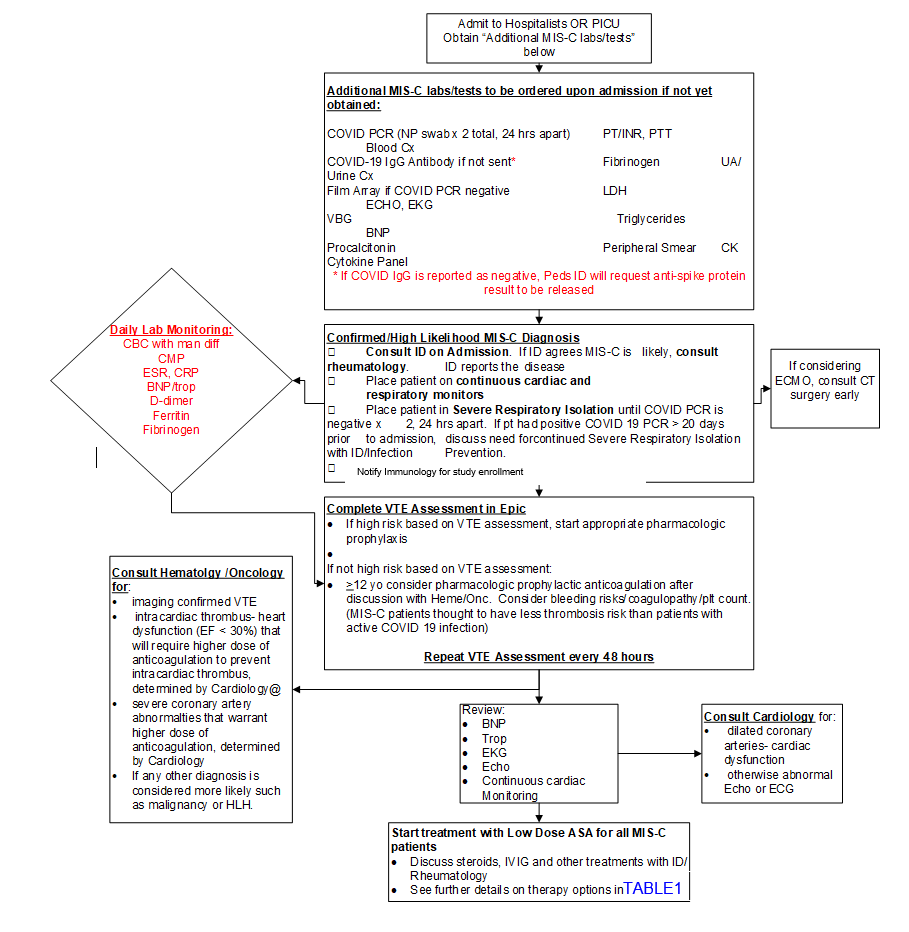


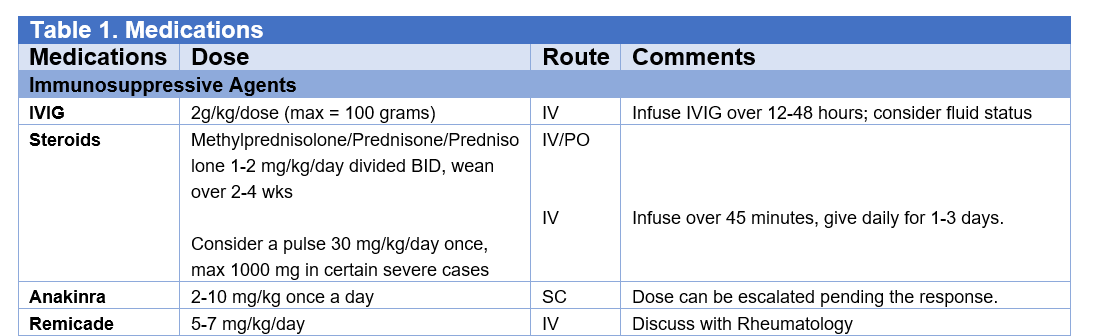

Supplement: Supplementary file 1 — Additional file 1: Appendix A. Institutional MIS-C Algorithm. [file 12969_2021_658_MOESM1_ESM.docx]
